# Case of Malformation of the Uterus, with Fatal Post-Partum Hemorrhage; Morbid Adhesion, and Partial Osseous Degeneration of the Placenta

**Published:** 1850-04

**Authors:** R. C. Hewett

**Affiliations:** Louisville, Kentucky


Art. II.—*Case of Malformation of the Uterus, with Fatal Post-partum Hemorrhage; Morbid Adhesion, and partial Osseous Degeneration of the Placenta.* By R. C. Hewett, M. D., of Louisville, Kentucky.

About four o'clock in the morning of Thursday, the 28th of February last, I was called to go about two miles out, to attend Mrs. T., in labor with her first child. I

arrived at her residence about 5 o'clock, A. M., and found a rather short, very fleshy but otherwise symmetrically proportioned lady, with an entirely healthy appearance and somewhat florid complexion. The mother of Mrs. T., a most sensible and estimable lady, was present and informed me, that this, her only daughter, and three sons, were all that survived of her fourteen children; that her first labor occupied three days; that she had had twins several times, but that this daughter was not a twin.

Mrs. T. was nineteen years of age, had been married three years, and was now pregnant for the first time—up to the time of marriage, and for some months after, her health was uninterruptedly good, and the catamenia continued regular in all respects, until the period of pregnancy. About eighteen months previous to the latter event, she became the subject of prolapsus uteri, and suffered greatly with pains in the back and left side. These pains continued to harass her up to the time of delivery. Until pregnancy occurred she weighed only one hundred and twenty-seven pounds, and at the time I saw her, in labor, her weight, she informed me, was one hundred and eighty pounds. By calculation, made at the time, I found that two hundred and eighty days had elapsed since her last catamenial day. At the first onset of pain, about two hours before my arrival, the membraneous sac had given way, and the liquor amnii had escaped. On examination I found the os uteri dilated to about the size of a dollar, and as I then concluded, the head presenting, but subsequent explorations disclosed a breech presentation, with the sacrum regarding the left acetabulum; it must have been the sacrum of the fetus, presenting at first, with which my finger came in contact. The bowels and bladder had been sufficiently evacuated, the pelvis had ample capacity, and every thing seemed favorable for a much less tedious labor than it proved to be. During the forenoon, slight cutting pains continued to recur at very irregular intervals, and in the afternoon they almost entirely ceased. At 10 o'clock, P. M., the pains became somewhat more active but were very transient; the os tincæ was not more dilated than in the morning; the patient suffered severely with constant pain in the back and left side, particularly if she attempted to lie down; she complained of headache; her face was flushed and the pulse was full and strong. About sixteen ounces of blood were drawn from the arm, with marked abatement of all the unpleasant symptoms; but still she could not lie down without excessive pain in the back and side, and the night was passed without sleep. On Friday morning, the patient was in good spirits, with the skin moist, circulation quiet, and the os uteri slightly more dilated. The relatives began to manifest great anxiety and apprehension on account of the unexpected delay in the case, and I expressed a willingness to have the opinion of any medical friend in relation to it. Dr. Knight was accordingly sent for, and, after careful examination, he concurred with me in opinion, that everything seemed favorable, and that nothing more could be done, nor should be attempted, to expedite the process, beyond the administration of opiates to procure sleep, and an enema, if necessary, to relieve the bowels. During this day and the succeeding night, the pains were very feeble, with intervals sometimes of several hours; and although laudanum and afterwards morphine were given, the patient reached Saturday morning without more sleep than transient dozing, and had continued to sit in a large chair. Tea and toast had been taken several times. She was sufficiently cheerful; showed no signs of exhaustion; the dilitation had made sensible progress, and the pains continued as before. About noon on Saturday, an enema of castor oil and laudanum was administered, which acted sufficiently. During the afternoon the uterus acted much more efficiently, and with more regularity, but the pains were still unusually short. The breech was fairly engaged in the superior strait, by 7 o'clock, being a period of sixty-four hours since the commencement of labor. Still there were no signs of unusual exhaustion. She was induced to lie down, on the left side, and as the abdominal muscles were now brought into action, rapid progress was made, so that by 8 o'clock, the presenting part was passing through the vaginal orifice. Three or four pains more expelled the body as far as the shoulders, and then the expulsive efforts seemed to cease. I directed ergot ʒss. to be immediately given; brought down the arms, and as the pulsations of the cord were very indistinct, the head was gently extracted. The child was remarkably large, perfectly formed, and irrecoverably asphyxiated. An immediate effort was made to set up respiration; and in a few moments my hand was applied to the abdomen of the mother, and the uterus, (I then supposed the whole of it,) seemed to be moderately contracted, although the tumor appeared to be longer and more pointed than I had ever noticed in any former case. The pulse was sufficiently full and firm, and not unusually accelerated. The mother of my patient was requested to place her hand upon the uterus and to follow it down with moderate pressure as it might contract. She remarked that it felt like a sugar loaf. My attention was again directed to the child, with attempts to resuscitate it, and after a few minutes I returned to the mother. Only one slight pain had occurred, and on making an examination the placenta could not be felt. Moderate traction was applied to the cord, and it became evident, from the firm sensation communicated by the cord, and by its mounting up, when relaxed, that the placenta was firmly adhering, high up in the uterus. This surprised me, as the latter seemed to be so much contracted. The pulse, also, was now more accelerated than I had ever observed in this stage. A message was immediately dispatched into the city for medical assistance, to come with all possible speed. Up to this time, the mother of Mrs. T. had her hand pressed constantly upon the uterine tumor, as directed. There was no external hemorrhage to excite apprehension, and for the third time I made efforts to reanimate the child; and whilst thus engaged I heard Mrs. T. sigh and ask for a glass of water—I immediately went to the bed, placed my hand on the abdomen, and found the uterus much higher up than at the previous examination, but it still had the remarkable pointed peculiarity mentioned above; and now I was astounded by feeling another tumor, larger than the first, and below it, in the position the patient was lying, on the left side. For the moment I concluded this might be owing to some peculiar spasmodic contraction of the uterus. The pulse had become quick and weak, with hurried respiration and sighs; the face was bedewed with perspiration, and all the fearful evidences of internal hemorrhage were before me. Fifteen or twenty minutes had now elapsed since the birth of the child. Thirty grains of ergot, all I had, were directed to be given at once; my coat was instantly thrown off, and I proceeded to introduce my hand to extract the placenta, and induce contraction. On penetrating the body of the uterus, my hand encountered masses of coagulated blood, and passed up into a cavity, that at one point admitted it, with some slight difficulty, and which, beyond this, was expanded into a sac somewhat larger than my closed hand. But the placenta could not be found. On slowly withdrawing my hand this cavity contracted upon it, and I then felt the edge of a substance, presenting downwards, and a little to the right of the patient, which I at first supposed was the placenta partly detached, but soon found that this substance was incorporated with the anterior and posterior walls of the uterus. I then found the cord, and following it up, my hand passed off to the left, into another sac, in which I found a great quantity of coagula, and the placenta firmly adhering, with the exception of about five inches of its circumference, nearest the center of the uterus. The substance I had before noticed was now ascertained to be a dense, thick septum dividing the upper part of the womb into two distinct cornua. In their then expanded condition, the fundus of each sac was distant from the inferior edge of this septum about the length of my hand, or seven inches. I commenced inserting my fingers between the placenta and uterus, and another peculiarity was disclosed, in numerous hard, rough points studding the uterine surface of the placenta. The after-birth was adhering with remarkable tenacity, and to remove it, without endangering the integrity of the uterus, required much caution. After it was separated, pressure was applied to the fundus of the placental side of the womb, whilst one of the attendants made firm counter-pressure externally, but no effort at contraction was made. The abdomen was covered with cloths dipped in cold water, and water was freely poured upon it, but the uterus remained as inert as if severed from the body. In the mean time my poor patient was becoming very weak and faint, the extremities cold, pulse feeble and fluttering, and the face perfectly blanched. Brandy and laudanum had been administered frequently and freely. They were continued, and the head was lowered as much as possible. I determined to withdraw the placenta, and some of the clotted blood. This was done as speedily as possible, and my hand was again carried into the uterus. The right side had expanded again, and my knuckles were pressed against the fundus of each cavity, alternately. External pressure and cold water were still applied, but no contraction ensued. All the usual expedients, within my reach, had now been used in vain, and I directed powerful, external pressure to be made over both sides of the uterus, so as to bring, if possible, the anterior and posterior walls in contact, whilst I compressed the abdominal aorta, a short distance above its bifurcation, as firmly as I could with my fingers. By this expedient I hoped to gain, at least, some time. Those who have had to manage cases of flooding, in which an amiable and confiding patient, in the midst of healthy, vigorous and youthful life, may have been reduced, in a few moments, to the very verge of existence, and have witnessed a family, full of joyous anticipations, suddenly overwhelmed with the most fearful forebodings and uncontrollable anguish, may form some estimate of the awful sense of responsibility that had possession of me at this moment. And although this case was surrounded with anomalous features and difficulties, fully sufficient to make a fatal issue unfavoidable, they did not, at the time, make my mental suffering the less. An hour had passed since Mr. T. started into the city with my message, when Professor Rogers, generously sympathizing with my condition, and braving the very inclement night and perilous road, entered the room. If an angel had suddenly appeared to rescue me from drowning, the presence could not have been more acceptible. Although for some time I had held the aorta firmly compressed, and all the other appliances had been continued, my patient continued to sink, and when Dr. Rogers arrived, she lay as still and white, and almost as cold and pulseless as a statue of alabaster, and thus quietly expired. Dr. Rogers quickly introduced his hand, and at first thought the uterus was contracted, but when I requested him to direct his hand to the left, it passed high up into the placental side, which was still perfectly flaccid. The right side had probably contracted, in some degree, and into it, Dr. R.'s hand had at first penetrated. He at once pronounced it a case of malformed uterus. We made a hasty examination of the placenta, and points of ossification, extending over its uterine surface, were distinctly perceptible to the sight and touch.

To Dr. T. G. Richardson I am indebted for his valuable services in conducting the autopsy.

*Autopsy—fifteen hours after death.*—The body was found moderately rigid and perfectly exsanguinous. Not a drop of blood appeared, at any point, in the track of the scalpel. There was a subcutaneous layer of fat, fully two inches in thickness, covering the abdomen, and the muscles were remarkably attenuated. The uterus was partially contracted, and instead of a convex border at the fundus, presented a very marked depression. At the point of depression and for some distance downwards, the anterior wall evidently projected forwards; and to the touch, externally, a thick, firm mass was felt occupying that portion of the cavity. The whole organ, with its appendages, was carefully exsected.

The relations of the deceased, with a promptness that did great credit to their good sense and intelligence, permitted us to retain the uterus and placenta.

The uterus measures from the fundus to the os tincæ eight and a half inches; its greatest width, when lying flat on a board, is seven inches. A partial, fleshy partition extends antero-posteriorly and in a longitudinal direction in the cavity of the uterus; being attached to the middle of the fundus, and reaching downwards about two and a half inches, with a free crescentic margin. Its antero-posterior width is about two inches; its thickness, at the attached part above, half an inch, and it gradually becomes thiner to the free edge. It is not unlike the falx cerebri in shape.

When the uterus is distended with water, it appears exteriorly, in front, like the female breasts, if the nipples were removed, the division between the two sides being in appearance like that between the breasts.

In all other respects the uterus seems to be perfectly normal. The deciduous membrane lined both sides, as patches of it are still adhering to the internal surface of the right or unoccupied side.

The placenta is peculiar, only in the points of ossification on its uterine surface, and in a somewhat oblong shape, with one end broader than the other.

These specimens were presented to Professor Cobb for the medical museum of the University of Louisville.

*Remarks.*—This case was probably an imperfect effort at what is called a double uterus.

From the fact, that before death, the right sac was smaller than the left—at one point scarcely admitting my hand—and from noticing that the free edge of the septum was then looking considerably to the right, I infer, that the fetus, as well as the placenta, occupied exclusively the left side; thus forcing the septum to the right of the mesial line with which it now corresponds. I conceive that this partition was not endowed with the same facility for growth or development and rapid contraction as the rest of the uterus. Otherwise I can see no reason why, at full term, the point of the fundus corresponding to the attachment of the septum was not as high up, at least, as any other part of the uterine walls, instead of being depressed as it was, and, with the two sides of the uterus expanded into two cornua, reaching up fully seven inches above the lower edge of the septum, as I noticed at the time.

This abnormal condition of the womb interrupted the favorable completion of parturition in several ways. In the first place, the fetus and placenta being confined to the left side, had less space for their development than in ordinary cases, and probably produced unusual distention, which may have been the cause of the ultimate fatal inertia of this side, and of the firm adhesion and partial degeneration of the placenta.

The axis of the left side being in an oblique direction to that of the superior strait, must have made the contractions much less effective in dilating the os tincæ, and aided in producing the protracted labor. In this condition of the parts, which of course could not be anticipated, the position in which my patient was lying, on the left side, was certainly not the most favorable; as it increased the obliquity of the axis of the left side of the uterus. This position, in connection with the existing obliquity, by favoring the accumulation of blood in the left sac, probably caused, also, the hemorrhage to be internal.

The bifid state of the fundus of the womb, making two distinct sacs, completely deceived me when I first applied my hand to the abdomen, after the birth of the child; for, although I noticed a peculiarity in the shape of the uterine tumor, it seemed to be the entire organ, and its smallness was the means of misleading me, the more, as I concluded the uterus was so much contracted. From the time of the birth of the child, the left cavity, I have no doubt, was fully expanded and the hemorrhage that stealthily exhausted the life of my patient was already in progress.

The irregular, inefficient contractions that marked the case throughout, were probably owing to the septum, that acted as a band through the center of the uterus, crippling its movements, and preventing simultaneous contraction of all its parts.

Even if the placenta had not been adherent, and had, at once, been expelled, still, it appears to me, the presence of the septum would have acted somewhat like that of the placenta, in preventing sufficient contraction, and in inviting hemorrhage. As this septum was not developed in proportion with the growth of the walls of the uterus, it is fair to conclude it could not contract so readily, nor until after some considerable time would elapse.

The complete inertia of the left cavity cannot be accounted for by supposing a state of general exhaustion arising from the protracted labor, for the countenance, state of mind and pulse of the patient, gave nò indications of unusual fatigue, and the right side did contract, readily, when my hand was first carried into it. It might, perhaps, be considered as in "a state of stupor caused by sudden evacuation," after unusual distention.

The most usual effect of morbid conditions of the placenta, is imperfect development of the fetus, and its premature expulsion from the uterus. In this case, the fetus was perfectly formed and very large; was undoubtedly living a short time before birth, and the full term of gestation had been completed.

As to the cause of abnormal adhesions of the placenta, much uncertainty seems to exist. In Cazeaux's Midwifery, I find the following, which is probably the sum of all that is known on the subject. "According to most authors, they are owing to a fibrous transformation of the cellular filaments which hold the placenta and uterus together, whereby they acquire a degree of solidity sufficient to withstand the uterine forces. These adhesions have also been referred to the degenerations of the placental tissue itself, as well as to various osseous and calcareous concretions."

Various authors mention the fact of the occasional occurrence of cases of double uterus, and of several other irregularities in the formation of the female organs of generation. Dr. Churchill mentions the *uterus bicollis*, *uterus bicorporeus*, *uterus biangularis*, &c., and says: "it is remarkable that these malformations, which are owing to an arrest of development, appear to reproduce the analogous organs of lower classes of animals." "These congenital malformations are by no means very rare; Dr. Cassan collected forty-one examples, and many others have since been reported." I have not found any new cases reported for some years past.

Dr. R. Lee says, in all cases of this kind, "without a single exception, the uterine appendages have been simple, or have consisted of one ovarium and one Fallopian tube annexed to each cornu of the uterus, and not of two ovaria and two Fallopian tubes, as the term double uterus would seem to imply."

In the Med. Chir. Rev., vol. 19, the following case is recorded: "M. Moreau (Academy of Medicine, Paris) presented a very beautiful specimen of a uterus completely divided into two equal, lateral halves; each provided with a tube and ovary; each separated from the other by a double partition, and each having distinct necks and mouths into a single vagina. The woman died after an accouchement. The fetus was a male, and had been developed in the left cavity; and, therefore, not in accordance with the whimsical idea that the right ovary furnishes the male and the left the female germs."

Dr. Robert Lee says: "In the museum of the Royal College of Surgeons, in London, there is a specimen of bifid unimpregnated uterus, and another was preserved in the collection of Mr. Brookes, in which the fundus, cervix and os uteri were all divided by a thick septum.—Similar cases have been recorded by different writers.—Messrs. Lauth and Cruveilhier have reduced all the malformations of the uterus to the four following varieties:

1. Where the uterus and vagina are separated into two cavities by a septum running in the direction of the mesial line, while the external configuration presents nothing unusual.

2. Where the fundus and body of the uterus are divided into two cornua, the cervix, os uteri, and vagina remaining in the normal state.

3. Where the uterus is bifid, as above, while the cervix and vagina are divided by a septum.

4. Where the vagina forms a single canal, with a double os uteri."

An attempt has been made to explain cases of alleged superfetation by supposing the existence of a double uterus; but this theory seems to be overthrown by the case, reported by Dr. Lee himself, "of impregnated double uterus, in which an organized deciduous membrane, in the form of a shut sac, lined the unimpregnated cornu, and rendered superfetation and menstruation impossible."

It might be a subject of very interesting inquiry, how far these cases of imperfect double uterus may favor the production of twins. As these malformations seem to be a reproduction of corresponding organs of the lower classes of animals, it is not altogether unreasonable to suppose that some tendency to the multiparous nature of these classes, may, in such cases, exist.

